# New York College of Veterinary Surgeons

**Published:** 1895-08

**Authors:** 


### New York College of Veterinary Surgeons.

The session begins in the latter part of September next under conditions, present and prospective, such as the veterinary colleges of New York State never faced before.

It can be said truthfully that at no time has this school been better equipped, from a veterinary standpoint, than for the coming session. With the added strength of such well-known men as Professors Huidekoper, Hickman, Lellman, and Mandel—all peculiarly well-equipped for the departments over which they will preside; with the new quarters, more nearly completed than a year ago and better equipped; with an enlarged curriculum and a more thoroughly graded course, the New York College should do its best work this year, all of which should tend to a higher standard of veterinary scholarship.

The course has been lengthened to cover the full period of six months, and the entrance examination has been planned to conform with the Board of Regents' Bill.

We note with pleasure the publishing of the requirements for membership in the United States Veterinary Medical Association—a proper and just recognition of the high place this organization holds in the good opinion of the profession. The admission of the alumni to lectures and clinics, on payment of a matriculation fee only, is a good movement.

